# Development of a loop-mediated isothermal amplification (LAMP) assay for the identification of the invasive wood borer *Aromia bungii* (Coleoptera: Cerambycidae) from frass

**DOI:** 10.1007/s13205-020-02602-w

**Published:** 2021-01-19

**Authors:** Domenico Rizzo, Nicola Luchi, Daniele Da Lio, Linda Bartolini, Francesco Nugnes, Giovanni Cappellini, Tommaso Bruscoli, Chiara Salemi, Raffaele V. Griffo, Antonio P. Garonna, Elisabetta Rossi

**Affiliations:** 1 Laboratory of Phytopathological Diagnostics and Molecular Biology, Plant Protection Service of Tuscany, Via Ciliegiole 99, 51100 Pistoia, Italy; 2 Institute for Sustainable Plant Protection, National Research Council (IPSP-CNR), Via Madonna del Piano 10, Sesto Fiorentino, 50019 Florence, Italy; 3 Department of Agriculture, Food and Environment, University of Pisa, Via del Borghetto 80, 56124 Pisa, Italy; 4 Institute for Sustainable Plant Protection, National Research Council (IPSP-CNR), P.le Enrico Fermi 1, 80055 Portici, Italy; 5 Plant Protection Service of Campania, Centro Direzionale, Isola A6, 80124 Naples, Italy; 6 Department of Agricultural Sciences, University of Naples Federico II, Via Università 100, 80055 Portici, Italy

**Keywords:** Red-necked longhorn beetle, Invasive pest, Rapid diagnostic tool, Phytosanitary survey

## Abstract

The red-necked longhorn beetle *Aromia bungii* (Faldermann, 1835) (Coleoptera: Cerambycidae) is native to east Asia, where it is a major pest of cultivated and ornamental species of the genus *Prunus*. Morphological or molecular discrimination of adults or larval specimens is required to identify this invasive wood borer. However, recovering larval stages of the pest from trunks and branches causes extensive damage to plants and is timewasting. An alternative approach consists in applying non-invasive molecular diagnostic tools to biological traces (i.e., fecal pellets, frass). In this way, infestations in host plants can be detected without destructive methods. This paper presents a protocol based on both real-time and visual loop-mediated isothermal amplification (LAMP), using DNA of *A. bungii* extracted from fecal particles in larval frass. Laboratory validations demonstrated the robustness of the protocols adopted and their reliability was confirmed performing an inter-lab blind panel. The LAMP assay and the qPCR SYBR Green method using the F3/B3 LAMP external primers were equally sensitive, and both were more sensitive than the conventional PCR (sensitivity > 10^3^ to the same starting matrix). The visual LAMP protocol, due to the relatively easy performance of the method, could be a useful tool to apply in rapid monitoring of *A. bungii* and in the management of its outbreaks.

## Introduction

*Aromia bungii* (Faldermann, 1835) (Coleoptera: Cerambycidae), the red-necked longhorn beetle, is an important pest of fruit and ornamental plants of the genus *Prunus*, both in native areas of east Asia and in newly invaded areas of Europe and Japan (EFSA 2019; EPPO 2020; CABI 2020). *A. bungii* can infest healthy or weakened host species and complete several overlapping generations in the same tree (Ma et al. 2007). The larvae bore galleries in the trunk and main branches, causing structural weakness, dieback, and finally tree death. Biological parameters of *A. bungii* evaluated in the Italian population showed remarkable fertility and longevity (Russo et al. 2020).

*A. bungii* is in the list of priority pests in the European Union (EU 2019) and quarantine measures have been applied in Germany and Italy to eradicate this invasive pest (Hörren 2016) or to contain the risk of further outbreaks (Carella 2019). These quarantine measures can have a strong impact on nurseries and farmers.

Early detection supported by rapid diagnostic protocols can help to identify the presence of *A. bungii* on plants irrespectively of the developmental stage of the pest, so that the efficacy of the phytosanitary monitoring in the field and at points of entry is enhanced. In the latter case, possible import delays can be avoided (Blaser et al. 2018; Poland and Rassati 2019).

The high specificity and sensitivity of DNA-based technologies allows the detection of harmful organisms even at low concentrations of DNA extracted from plant tissues (Aglietti et al. 2019; Rizzo et al. 2020a). Among the most versatile, sensitive and specific methods, loop-mediated isothermal amplification (LAMP) can be used as a field-friendly and cost-effective diagnostic tool (Notomi et al. 2000, 2015). Several LAMP tests have been used both in the field and in laboratories, in particular for human and animal diseases (Lucchi et al. 2010), in food safety controls (Abdulmawjood et al. 2014), as well as in identifying plant pathogens (Aglietti et al. 2019; Luchi et al. 2020; Blaser et al. 2018) and invasive insect pests (Huang et al. 2009; Hsieh et al. 2012; Fekrat et al. 2015; Przybylska et al. 2015; Ide et al. 2016a, b; Blaser et al. 2018; Sabahi et al. 2018; Rizzo et al. 2020b). LAMP is a highly specific and robust identification method for species with previously known DNA or RNA sequences and suitable for on-site application because it can be performed in a laboratory-free environment after minimal training (Kogovšek et al. 2015).

This paper presents a reliable and sensitive diagnostic test for the rapid diagnosis of *A. bungii* frass using the LAMP technique. The quality of this method is compared to the conventional PCR end point method and a qPCR protocol recently developed for the identification of *A. bungii* from frass (Rizzo et al. 2020a).

## Materials and methods

### Biological samples

**Fig. 1:**
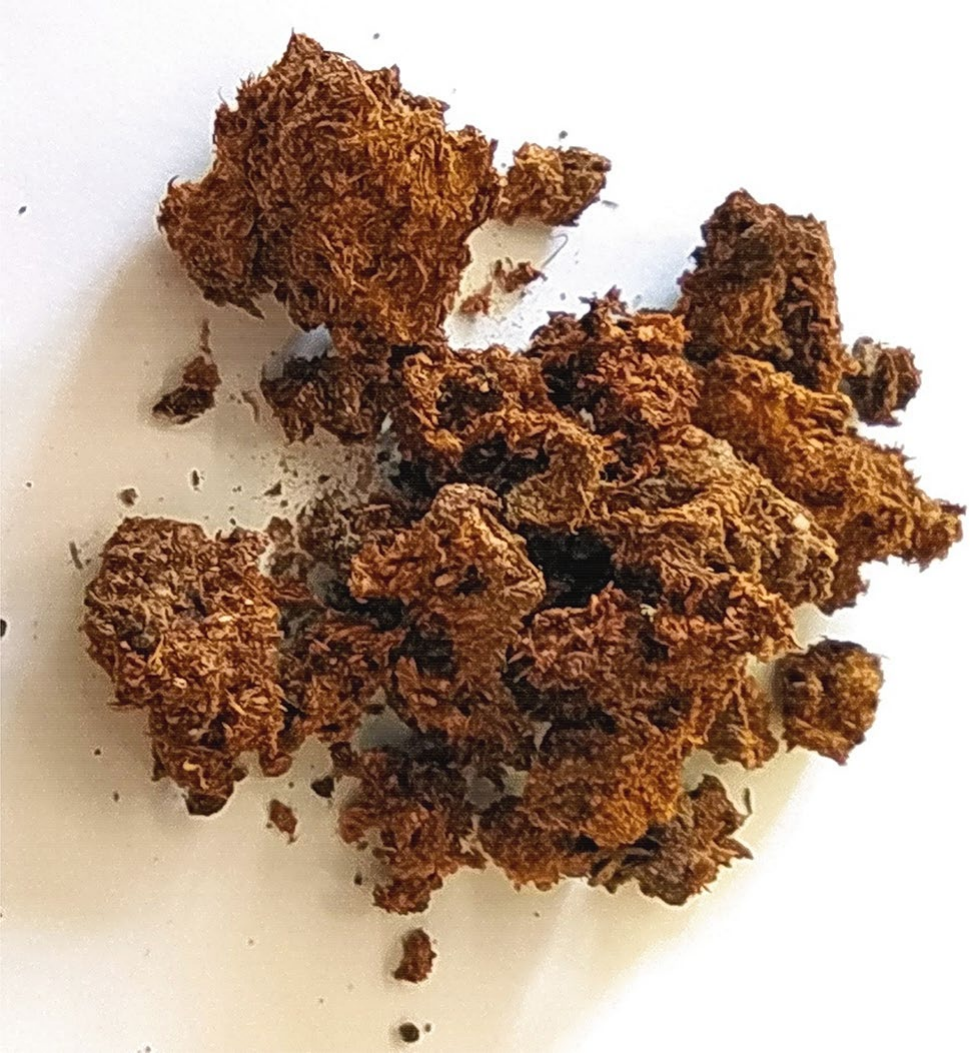
Sample of *Aromia bungii* frass collected in the field

The target samples included adults, larvae, and frass of *A. bungii*. Adults and larval specimens were supplied by the Department of Agricultural Sciences of the University of Naples “Federico II” and the Plant Health Service of the Campania region. In some farms situated in the pest outbreak area around Naples (Campania, Italy), where *A. bungii* is considered as established (Carella 2019), frass samples (Fig. 1) were collected at the trunk base of *Prunus* plants and individually labeled as in Rizzo et al. (2020a).

The non-target samples consisted of a set of DNA samples from the entomological biomolecular collection of the phytopathological laboratory of the Phytosanitary Service of the Tuscany Region. The non-target DNA samples were listed in a previous paper (Rizzo et al. 2020a) and included a total of 62 samples belonging to 26 species. They were used for testing the diagnostic specificity of the protocols. The non-target samples included, depending on the species, adults and/or larval specimens and frass samples in the case of some xylophagous species. Among the non-target species, a subset of six xylophagous species producing frass (*Anoplophora chinensis* (Forster), *An. glabripennis* (Motschulsky), *Cerambyx cerdo* Linnaeus, *Cossus cossus* Linnaeus, *Sesia* sp. Fabricius, and *Zeuzera pyrina* Linnaeus) was chosen and DNA was extracted de novo from their frass for this study. These DNA samples will be hereafter be referred to as non-target frass samples.

### DNA extraction

The DNA extraction procedure was the same for real-time and visual LAMP protocols but had some changes in relation to the matrix (frass or larvae/adults). The extraction was carried out on *A. bungii* frass and larvae or adults following the CTAB extraction method suggested in Li et al. (2008) with slight modifications. Specifically, in the extraction from insect frass, about 1 g of matrix was homogenized in a 10-mL stainless steel grinding jar along with a TissueLyzer (Qiagen, Hilden, Germany) for 10 s at 2000 opm. Each larva/adult was ground and homogenized individually using nylon mesh U-shaped bags (Bioreba, Reinach, Switzerland). Variable volumes (10 mL for insect frass and 1 mL for larvae) of 2% CTAB buffer (2% CTAB, 1% PVP-40, 100 mM Tris–HCl, pH 8.0, 1.4 M NaCl, 20 mM EDTA, and 1% sodium metabisulfite) were added immediately after grinding.

A volume of 0.5–1 mL of lysate was then incubated at 65 °C for 10 min, 1 volume of chloroform was added, stirred by inversion and TissueLyzer centrifuged at 13,000 rpm for 10 min. An aliquot of 600 µL was then taken from the supernatant and an equal volume of isopropanol was inserted, mixed by inversion and centrifuged at 13,000 rpm for 5 min. The resulting pellet was dried by speed vacuum (Eppendorf, Milan, Italy) for 5 min, then resuspended in 100 µL of sterile, ultra-pure water and incubated at 65 °C for 5 min and used for LAMP/qPCR/conventional PCR reactions immediately or stored at − 20 °C until use.

This extraction protocol was used on *A. bungii* samples (larvae and frass) and non-target frass samples in triplicate. The amount of DNA (ng/μL) and the *A*_260/280_ ratios were evaluated for each sample using the QIAxpert spectrophotometer (Qiagen, Hilden, Germany). To detect biological traces of insects (feces, etc.) in the frass samples, the quality of the extracted DNA was estimated using a dual-labeled qPCR targeting a highly conserved region of the 18S rDNA (Ioos et al. 2009).

LAMP reaction targeting the cytochrome c oxidase subunit I (COI) gene was also performed on frass samples to assess the amplifiability of the extracted DNA from wood (Tomlinson et al. 2010b).

### Design of *A. bungii* LAMP and conventional PCR end point primers

In the LAMP reaction, six primers (F3/B3, FIP/BIP and LoopF/LoopB) were designed to specifically target a fragment of the cytochrome oxidase subunit I (COI) gene of *A. bungii* (accession n. KF737790). The primers were designed using the LAMP Designer software (OptiGene Limited, Horsham, UK) and synthesized by Eurofins Genomics (Ebersberg, Germany). The sequences of the primers are shown in Table 1.

**Table 1:** *Aromia bungii*–The loop mediated isothermal amplification (LAMP) method primers designed in this study. For each primer, the nucleotide position related the the reference sequence is reported. The product sizes are 254 bp (F3–B3) and 165 bp (F2–B2). The reference sequence is KF737790.

Primer name	Length (nt)	Sequence 5′–3′	Nucleotide position
Abungii_F3	20	CTGGAACTGGATGAACAGTT	365–384
Abungii_B3	20	AATGGCTCCTGCTAATACTG	618–599
Abungii_FIP(F1c + F2)	23 + 21	AATTAACGGCACCGAGGATTGAA CCATGGAGGATCTTCAGTAGA	490–468 411–431
Abungii_BIP(B1c + B2)	25 + 22	ACTGTTATTAATATGCGCCCTTCCG CTGTAATAACAACAGCTCACAC	499–523 575–553
Abungii_LoopF	25	GAGATTCCTGCTAGATGAAGTCTAA	467–443
Abungii_LoopB	24	GGATAAGTCCAGATCGTATACCTT	524–547

**Fig. 2:**
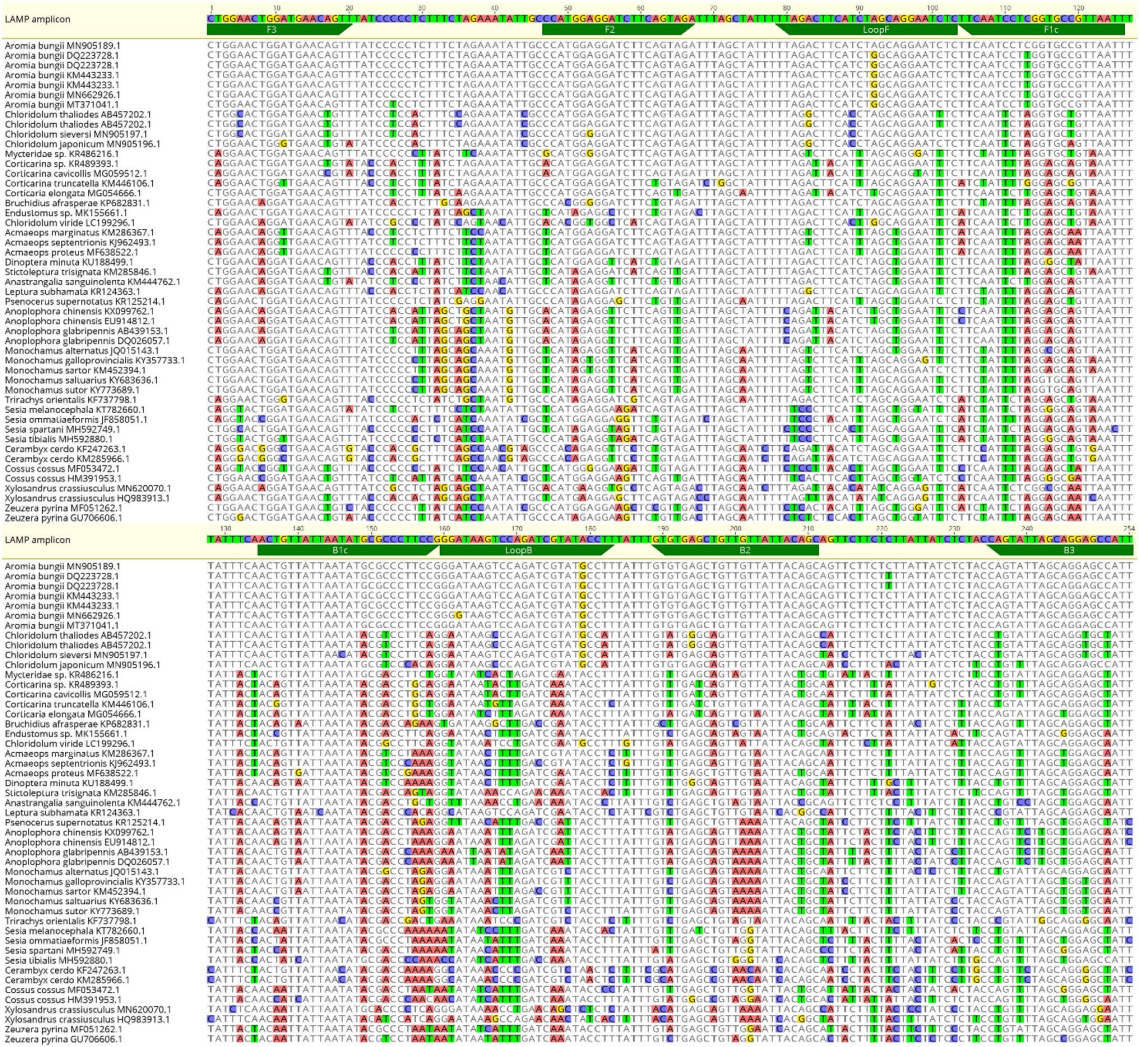
Alignment of the LAMP amplicon of *A. bungii* and the sequences belonging to the most taxonomically related species (included the non-target xylophagous species used in the assays) present in GenBank. The reference sequence is the *A. bungii* LAMP amplicon (yellow areas); the correspondent primers are reported in blue. The nucleotides which differ from the reference sequence are highlighted with different colors according to the specific base

The specificity of the primers was further tested using BLAST^®^ (Basic Local Alignment Search Tool: http://www.ncbi.nlm.nih.gov/BLAST; Altschul et al. 1990). *A. bungii* LAMP homologous sequences were downloaded from GenBank and used for alignments to test the in silico specificity of the designed primers. The alignments were performed using the MAFFT software implemented in Geneious 10.2.6 (Kearse et al. 2012), set with the default parameters (Fig. 2).

To evaluate and compare the analytical sensitivity, specificity and reliability of the developed real-time and visual LAMP protocols, conventional PCR (end point) assays for the diagnosis of *A. bungii* were designed (Table 2) using the OligoArchitect™ Primers and Probe Online software (Sigma-Aldrich, St. Louis, USA) with the following specifications: a 100–380 bp product size, a Tm (melting temperature) of 55–65 °C, primer length of 18–26 bp, and absence of secondary structure when possible.

**Table 2:** Basic parameters of the methods compared to the LAMP assay

Primers	Sequence (5′–3′)	Length (bp)	Annealing (°C)	Protocol	Reference
Abungii_F3	(Outermost primers of LAMP protocols in this study)	254	60	End-point PCR	This study
				qPCR SYBR Green	This study
Abungii_B3					
Abungii_285_F	CAGCAGTTCTTCTTTTATTATC	199	58	qPCR Probe	Rizzo et al. (2020a)
Abungii_484_R	GGTGTCCAAAGAATCAAA				
Abungii_309_P	FAM-TACCAGTATTAGCAGGAGCCATTACG-BHQ1				
Abungii_436F	TAACTTCCGTCTATTAGATGTA	157	55	qPCR SYBR Green	Rizzo et al. (2020a)
Abungii_592R	GCTAACTTGGTTGATTCG				
Abungii_51_F	TCTATACTTTATCTTCGGTGCATGA	318	55	End-point PCR	This study
Abungii_368_R	CCAGCACCCCTTTCTACGATT				
Abungii_28_F	ACCAACCATAAAGATATTGGAACTC	462	54	End-point PCR	This study
Abungii_489_R	ATTAACGGCACCGAGGATTGA				

### LAMP assay and conventional PCR end point optimization

*Real-time LAMP.* The real-time LAMP reactions were performed using the Isothermal Master Mix (ISO-001) produced by OptiGene Limited (Horsham, UK) on a CFX96 thermocycler. Each isothermal reaction was performed in duplicate, in a final volume of 20 μL and using 2 μL of DNA. Negative controls (NTC—no template control) were included for each reaction. At the end of the LAMP reactions, a melting curve was generated by increasing the temperature from 65 to 95 °C with a 10-s interval every 0.5 °C (Abdulmawjood et al. 2014). In real-time LAMP amplification, raw data were analyzed using CFX Maestro v. 1.0 (Biorad, Berkeley, CA, USA). Real-time LAMP products were checked on a 1.7% agarose gel stained with Gel Red (Biotium, Fremont, CA, USA).

The LAMP protocol optimization considered the following variables: isothermal amplification time, primer concentration and annealing temperature through a thermal gradient. Once the LAMP reaction had been optimized, the reactions were carried out using a second portable thermocycler, Genie^®^ II (Optigene, Ltd, Horsham, UK) to evaluate their reproducibility.

**Fig. 3:**
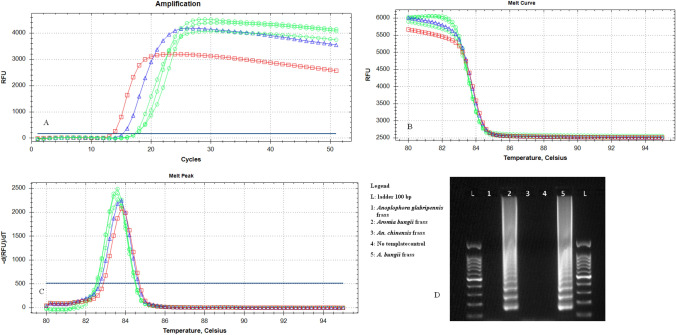
Real-time LAMP amplification curves (**a**) and melting curve (**b**) and peak (**c**) from larva (squares), adult (triangles), and frass (circles) of *Aromia bungii* and NTC (no template control) (diamonds); **d** agarose gel showing the amplification product

The optimal reaction mix for the real-time LAMP assay consisted of 10-μL Isothermal Master Mix OptiGene (ISO-001), 0.2 μM of F3/B3, 0.4 μM of LoopF/LoopB, 0.8 μM of FIP/BIP and 2 μL of template DNA (5 ng/μL) in a final volume of 20 μL. The melting peak for *A. bungii* samples was 83.5 ± 0.5 °C (Fig. 3).

*Visual LAMP.* To develop an alternative and easy-to-use protocol to detect the *A. bungii* DNA from collected samples, a visual LAMP approach based on the primers designed for the real-time LAMP assay was also tested. The Bst 3.0 DNA polymerase kit (New England Biolabs Ltd., UK) was used for LAMP reactions on *A. bungii* DNA from frass with the same six LAMP primers used in the real-time LAMP test. Hydroxynaphthol Blue (HNB) was included in the reaction mixture (Goto et al. 2009) and the color change (from purple to blue) was evaluated at the end of the reaction.

To optimize the visual assay conditions, the same parameters considered for the real-time LAMP were assessed. The following reagents were optimized in their quantities and/or concentrations: buffer, dNTPs, Betaine, MgSO4, HNB and primer concentration and Bst 3.0 DNA polymerase. The reaction was performed at 65 °C for 30 min, followed by an additional cycle of 80 °C for 2 min. Isothermal amplifications were analyzed with a QIAxcel Capillary Electrophoresis System (QIAgen, Valencia, CA, USA) with the inclusion of a 25 bp DNA marker. The QIAxcel system uses ScreenGel software, which determines the base pair number of each amplicon in individual amplification reactions.

**Fig. 4:**
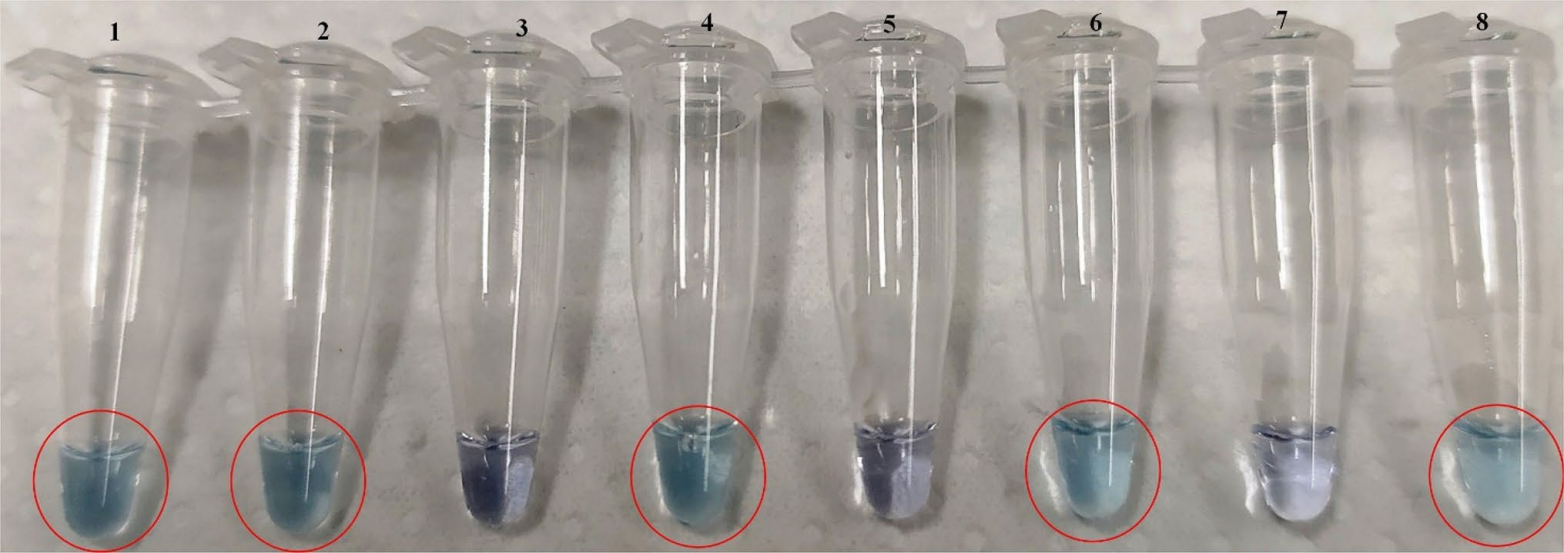
Visual LAMP reaction tubes visualized by means of HNB (Hydroxynaphthol Blue) coloration. Positive samples are blue, and negative are purple. The assay was performed on DNA extracted from (left to right): tubes 1 and 2, *Aromia bungii* larvae; tube 3, *Anoplophora chinensis* frass; tube 4, *A*. *bungii* frass; tube 5, *An. glabripennis* frass; tube 6, *Aromia bungii* frass, tube 7, *C. cossus* frass and tube 8, *A. bungii* frass. The circles show the tubes containing *A. bungii* samples

The 20-μL optimal visual LAMP reaction mixture consisted of 2 μL of Isothermal Buffer 10X, 0.6 mM of dNTPs, 2 mM of MgSO_4_, 0.15 mM of HNB, 0.2 M of Betaine, 0.32 U/μL of Bst 3.0 and final concentrations of the LAMP primers equal to 0.2 μM for F3/B3, 0.4 μM for LoopF/LoopB, 0.8 μM for FIP/BIP. 2 μL of DNA template (5 ng/μL) was considered. The visual LAMP protocol was carried out on *A. bungii* and non-target DNA from frass of *An. chinensis*, *An. glabripennis* and *C. cossus* (Fig. 4).

*Conventional PCR.* The conventional PCR reactions were performed in 20-μL reaction volumes containing 1X Master Mix PerfectTaq Hot Start 5Prime (Eppendorf, Milan, Italy), 0.4-µM forward and reverse primers, and 2 μL of DNA template in a MyCycler thermocycler (Biorad, Berkeley, CA, USA). Cycling conditions consisted of 3 min at 94 °C, followed by 40 cycles of 94 °C for 30 s, annealing (see Table 2) for 30 s and 72 °C for 45 s, with a final extension step of 7 min at 72 °C. PCR products were visualized on a 1.7% agarose gel using a 10-μL aliquot of PCR reaction or using a QIAxcel Capillary Electrophoresis System.

### Performance characteristics of the LAMP assay

Sensitivity, specificity and accuracy of the real-time and visual LAMP assays were evaluated after the optimization of the LAMP protocols on DNA samples of target and non-target species (62 samples belonging to 26 species). Samples with a time amplification value (Tamp, min:s) (Aglietti et al. 2019) greater than 30 min were not considered. In the visual LAMP, the diagnostic specificity was verified by the naked eye assessing the color change of the reaction mixture. These parameters were calculated according to the EPPO standards on diagnostics PM7/76-4 (EPPO 2017) and PM7/98-4 (EPPO 2019).

### Blind panel validation of the assays

A blind panel test was performed on six frass samples of *A. bungii*, two of *Anoplophora chinensis*, two of *An. glabripennis* and two of *C. cossus*. The test was carried out in two different laboratories (IPSP-CNR, Sesto Fiorentino, Italy and the Laboratory of the Plant Protection Service of Tuscany, Pistoia, Italy) applying the above-mentioned LAMP (real-time and visual) protocols. All DNA samples had been diluted at a final concentration of 5 ng/µL. Samples were tested in duplicate; negative controls (NTC—no template control) were included. Based on the blind panel results, the true positives, false negatives, false positives and true negatives were evaluated according to the EPPO requirements outlined in PM7/76-4 (EPPO, 2017) and PM7/98-4 (EPPO 2019).

### Repeatability and reproducibility

The repeatability and reproducibility tests were carried out on ten samples of *A. bungii* DNA extracted from frass. The intra-run variation (repeatability) and inter-run variation (reproducibility) were estimated by standard parameters, such as the average Tamp and standard deviation (SD). Ten samples in triplicate, diluted to a final concentration of 5 ng/µL, were tested in two separate series for repeatability. The reproducibility of each protocol was assessed in the same way as carried out for the repeatability by comparing the data of two series of samples by two different operators on different days (Dhami et al. 2016; Koohkanzade et al. 2018).

### Limit of detection (LoD)

For each methodology used in the experimental design, LoD was estimated using a tenfold 1:4 serial dilution using an “artificial” frass DNA (100 ng/µL), obtained by adding frass of another species (*An. glabripennis*, in this case) with 10 ng/µL of *A. bungii* DNA from larvae. All experiments were conducted in triplicate. To evaluate the influence of the initial matrix in defining the analytical sensitivity of the method under examination, the LoD verified with pure larva DNA extract and DNA extract from *A. bungii* artificial frass were compared. The comparison between the LoDs of the end point PCR and LAMP protocol was carried out by electrophoretic runs in 1.7% agarose gel stained with Gel Red (Biotium Inc., Fremont, CA, USA). In parallel, a QIAxcel Capillary Electrophoresis System (QIAgen, Valencia, CA, USA) was used with the inclusion of a 25-bp DNA marker.

### Comparison with conventional PCR and qPCR (SYBR Green)

To compare the sensitivity and performance of the assay, frass was used as the matrix with other molecular techniques, traditional end point PCR and qPCR (both hydrolysis probe and SYBR Green), performed with the parameters reported in Table 2.

The F3 and B3 primers, which are "external" to the ones used in the LAMP assay, were used in both conventional PCR and in qPCR SYBR Green.

## Results

### Nucleic acid extractions from frass and insects

The amplifiability of the DNA extracted from target and non-target frass samples (Table 3) gave satisfactory results. The Tamp average value of COX gene (LAMP protocol) was 12.3 ± 2.4 (min). The verification of amplifiability with the qPCR probe on insect extracts showed average values of Cq equal to 18.64 ± 3.6.

**Table 3:** Quantification of DNA extract from frass of *Aromia bungii* and non-target frass samples

Species	DNA concentration (ng/µL) (mean ± SD)	*A*_260/A280_ ratio (mean ± SD)
*Aromia bungii*	85.10 ± 4.00	1.94 ± 0.16
*Anoplophora chinensis*	101.02 ± 2.60	1.84 ± 0.14
*Anoplophora glabripennis*	94.14 ± 5.20	1.82 ± 0.18
*Cerambyx cerdo*	76.56 ± 2.30	1.76 ± 0.20
*Cossus cossus*	89.24 ± 2.30	1.86 ± 0.11
*Sesia* spp.	68.63 ± 3.20	2.01 ± 0.17
*Zeuzera pyrina*	62.25 ± 2.90	1.88 ± 0.18

### Diagnostic sensitivity, specificity, and accuracy of the LAMP assay

None of the tests carried out on target and non-target samples showed any non-specific amplification, and only *A. bungii* produced amplification curves. A unique peak at 83.5 ± 0.5 °C, resulting from the melting curve analysis, was visualized for each *A. bungii* sample, regardless of the starting matrix and confirming the specificity of the real-time LAMP assay. In the case of the visual LAMP assay, only *A. bungii* samples (adult, larvae, and frass) were detected by the LAMP reaction, while none of the non-target samples (62 samples) was amplified. For both protocols, diagnostic sensitivity, diagnostic specificity, and relative accuracy were 100%.

The end point PCR protocols designed to compare the analytical sensibility (LoD) were also assayed on the same target and non-target samples, showing a diagnostic specificity of 100%, as in the LAMP assay developed in this study.

### Blind panel validation of the assay

The blind panel test performed using the real-time and visual LAMP protocols showed the amplification only of the *A. bungii* frass samples, with a mean Tamp value equal to 18.21 ± 0.42 min in the case of real-time LAMP, whereas the non-target frass samples were not amplified. The specificity, sensitivity and accuracy of the data were 100%. In both laboratories, the results obtained with real-time and visual LAMP were the same. Only the *A. bungii* frass samples amplified, whereas there was no amplification of the DNA samples extracted from the frass of the xylophagous species used as comparison (non-target frass samples).

### Repeatability and reproducibility of the diagnostic methods

In terms of repeatability, the Tamp values varied from 10.12 to 13.30 min with a mean value of 10.90 ± 1.20 min, and an average CV% of 11.04.

The standard deviation (SD) of the two replicates of the same protocol ranged between 0.06 and 3.65. In terms of reproducibility, the values ranged between 0.10 and 7.24 (Table 4).

**Table 4:** Repeatability and reproducibility of real-time assays evaluated as Cq ± SD values

Sample	Real-time LAMP amplification		
	Repeatability		Reproducibility
	Assay 1	Assay 2	
1	17.37 ± 0.06	16.48 ± 1.20	16.52 ± 1.26
2	19.12 ± 0.66	18.09 ± 2.13	17.62 ± 1.46
3	24.28 ± 3.65	19.16 ± 3.59	21.74 ± 7.24
4	18.85 ± 0.65	18.60 ± 0.29	19.06 ± 0.36
5	17.77 ± 2.03	17.36 ± 1.45	18.79 ± 0.58
6	17.41 ± 0.83	17.58 ± 0.59	16.99 ± 0.24
7	16.74 ± 1.25	16.29 ± 0.62	17.18 ± 0.63
8	18.58 ± 2.18	16.57 ± 1.52	18.11 ± 0.66
9	16.87 ± 2.05	16.28 ± 1.22	17.73 ± 0.83
10	17.66 ± 0.83	17.59 ± 0.74	18.18 ± 0.10

### Limit of detection (LoD) of the LAMP assay and comparison with conventional PCR and qPCR

The LoD was obtained both for the real-time LAMP assay and for the visual LAMP. For the real-time assay, the LoD was 0.61 pg/µL, with a Tamp value of 24.36 ± 0.90 min. For the visual LAMP assay, the LoD was the same as for the real-time LAMP assay.

Table 5 compares the LoD values obtained in the different techniques. The data assigned to the PCR protocols (probe for hydrolysis and SYBR Green) (Rizzo et al. 2020a), have been omitted in this table.

**Table 5:** LoD assay based on artificial frass of *Aromia bungii* using 1:4 serial dilutions (ranging from 10 ng/µL to 2.38 fg/µL) and the real-time LAMP protocol. For each dilution different PCR methods were evaluated. The average Cq/Tamp ± standard deviation (SD) was equal to the average of the three threshold cycles of each dilution (Cq/Tamp) ± SD. In the case of qPCR, Cq values above 35 were considered as negative results. (1) PCR end point (F3/B3, this study); (2) PCR end point (28F/489R, this study); (3) qPCR SYBR Green (F3/B3 this study). The ± symbol in the visual LAMP column indicates an uncertain result

Dilutions 1:4	Diagnostic method				
	Real-time LAMP	Visual LAMP	PCR end point (1)	PCR end point (2)	qPCR SYBR Green (3)
	Tamp (min:s) mean ± SD	Positive (+)/negative (−)	Positive (+)/negative (−)	Positive (+)/negative (-)	Cq means ± SD
10 ng/µL	14.22 ± 1.49	+	+	+	18.99 ± 0.84
2.50 ng/µL	15.24 ± 1.68	+	+	+	21.02 ± 0.31
0.62 ng/µL	16.59 ± 1.88	+	+	+	22.82 ± 0.76
0.16 ng/µL	18.59 ± 1.38	+	+	+	24.60 ± 0.54
0.04 ng/µL	21.53 ± 3.07	+	+	+	26.93 ± 0.16
9.76 pg/µL	23.99 ± 0.73	+	–	–	29.43 ± 0.25
2.44 pg/µL	26.84 ± 1.76	+	–	–	32.31 ± 0.28
0.61 pg/µL	24.36 ± 0.90	**±**	–	–	33.44 ± 0.07
0.15 pg/µL	–	–	–	–	–
38.14 fg/µL	–	–	–	–	–
9.53 fg/µL	–	–	–	–	–
2.38 fg/µL	–	–	–	–	–

**Fig. 5:**
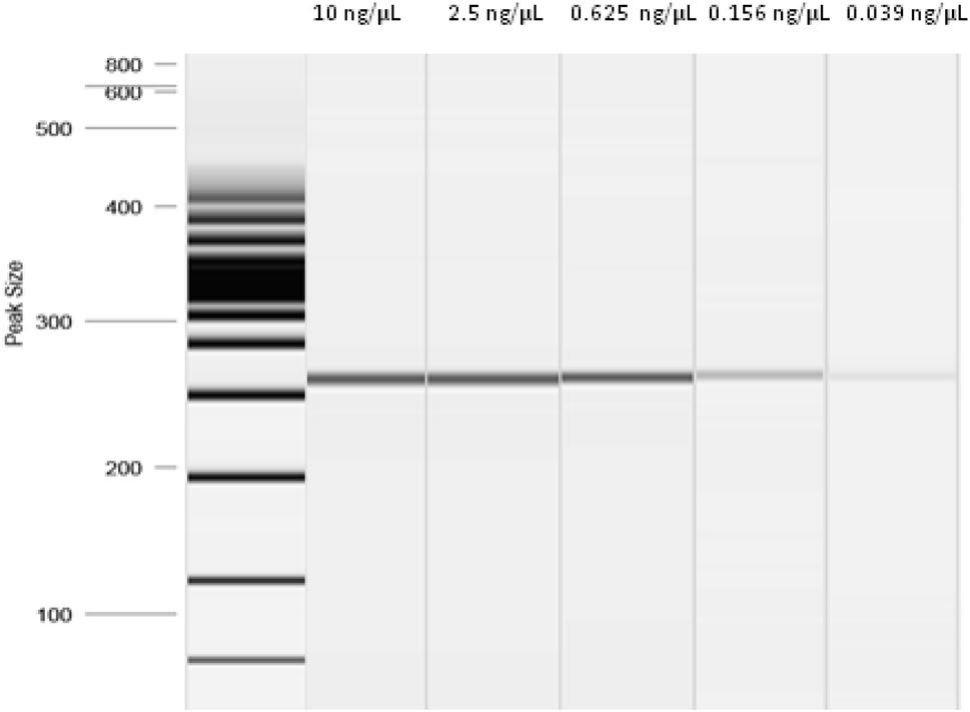
Electrophoresis capillarity with QIAxcel Capillary Electrophoresis System (Qiagen, Valencia, CA, USA). PCR end point with primers F3 and B3 on serial dilution 1:4 from frass artificial of *Aromia bungii*. The marker ranged from 25 to 3000 bp (Qiagen, Valencia, CA, USA)

**Fig. 6:**
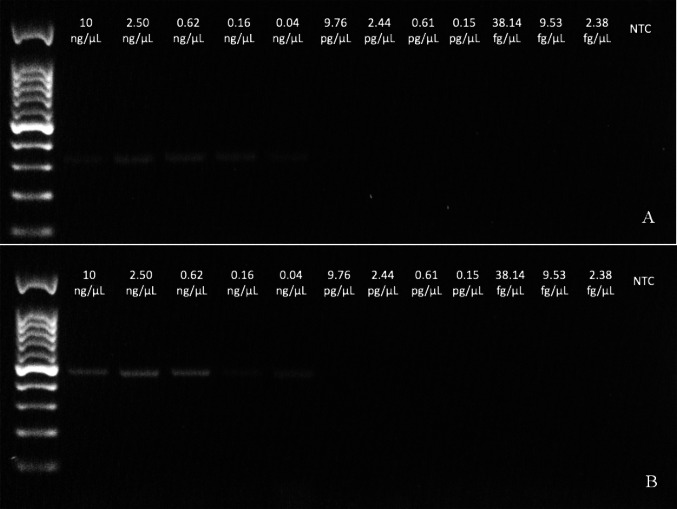
Agarose gels for PCR end point with 51F/368R primers (**a**) and with 28F/489R primers (**b**) on serial dilution 1:4 from artificial frass of *Aromia bungii*. The ladder weight was 100 bp (Genespin, Milan, Italy)

Figures 5 and 6 show the results of the electrophoretic runs carried out to compare the LoDs of the conventional PCR (end point) and LAMP, using 1.7% agarose gel stained with Gel Red and QIAxcel Capillary Electrophoresis System (Qiagen, Valencia, CA, USA), respectively.

The comparison of the analytical sensitivity according to the starting matrix (larva and artificial frass) provided the data shown in Table 6.

**Table 6:** Analytical sensitivity (LoD) between 1:4 serial dilutions of *Aromia bungii* larva DNA extract (10 ng–2.38 fg/ µL) and 1:4 serial dilutions of *Anoplophora glabripennis* artificial frass extract to which 10 ng of *A. bungii* larva extract was added. The ± symbol in the A. bungii visual LAMP column indicates an uncertain result

Dilutions 1:4	DNA extract from *A. bungii* larva		DNA extract from *An. glabripennis* artificial frass	
	Real-time LAMP	Visual LAMP	Real-time LAMP	Visual LAMP
	Tamp (min:s) avg ± SD	Positive (+)/negative (−)	Tamp (min:s) avg ± SD	Positive (+)/negative (−)
10 ng/µL	14.62 ± 0.34	+	14.22 ± 1.49	+
2.50 ng/µL	14.77 ± 1.81	+	15.24 ± 1.68	+
0.62 ng/µL	16.07 ± 0.15	+	16.59 ± 1.88	+
0.16 ng/µL	16.12 ± 1.30	+	18.59 ± 1.38	+
0.04 ng/µL	16.79 ± 0.81	+	21.53 ± 3.07	+
9.76 pg/µL	18.60 ± 1.73	+	23.99 ± 0.73	+
2.44 pg/µL	24.80 ± 10.78	+	26.84 ± 1.76	+
0.61 pg/µL	25.74 ± 13.00	+	24.36 ± 0.90	+
0.15 pg/µL	26.10 ± 4.20	+	n/a	–
38.14 fg/µL	32.18 ± 4.41	+	n/a	–
9.53 fg/µL	26.78 ± 0.46	**±**	n/a	–
2.38 fg/µL	n/a	–	n/a	–

## Discussion

Molecular tools for identifying quarantine insect pests are essential for managing outbreaks, especially in view of the setup of international shared diagnostic protocols (Augustin et al. 2012). Of these molecular methods, the LAMP technique (Tani et al. 2007; Tomlinson et al. 2010a; Moradi et al. 2014; Blaser et al. 2018; Panno et al. 2020) can be used for a direct diagnosis of insect specimens (adults or larvae), as well as for an indirect analysis of insect DNA present in residues deriving from the trophic activity (e.g., frass as in Kyei-Poku et al. 2020).

For frass samples, three critical points must be considered: (a) the paucity of insect DNA in these samples; (b) the presence of amplification inhibitors deriving from frass (Mitchell and Hanks 2009; Schrader et al. 2012; Strangi et al. 2013; Nagarajan et al. 2020; Rizzo et al. 2020a, b); and (c) the possibility DNA degradation over time or as an effect of frass exposition to environmental factors.

We used the LAMP method on *A. bungii* frass. Our results show that all three issues (a–c above) were overcome. In all samples, the DNA quantity was always suitable and amplifiable for the LAMP reactions, managing the co-extraction of inhibitors from the frass samples, and with an A_260/280_ ratio of between 1.8 and 2.0. However, the DNA amount extracted from adults and larvae of *A. bungii* was higher than in the frass samples, but with a higher variability in terms of concentration, probably related to the specimen size.

Our real-time LAMP protocol on frass gave good results in terms of specificity, especially given that *Aromia moschata*, a native species taxonomically related to *A. bungii* included in the non-target species assayed, did not respond to the amplification reaction (Rizzo et al. 2020a). The protocol was also sensitive and accurate, and overall, the reaction demonstrated its robustness when the test was performed on different thermocyclers and with different operators. The repeatability and reproducibility data showed SD values with a relatively high range (Teter and Steffen 2020), of variability (presumably due to the presence of a high quantity of PCR inhibitors in frass).

The use of LAMP based on a naked-eye detection system to determine the amplification result is becoming a routine approach in molecular diagnosis (Blaser et al. 2018). Our visual LAMP is a further simplification of the real-time LAMP technology as it does not require sophisticated instruments (which entail large investments, skilled personnel, and high management costs), is rapid, specific, sensitive and with a good accuracy, also compared to real-time LAMP. In addition, the limits of detection are identical to those of real-time LAMP (LoD of 0.61 pg/µL for the proposed techniques).

The analytical sensitivity of the LAMP (LoD) test compared with conventional PCR (28F/489R and 51F/368R) was more sensitive (> 10^3^) to the same starting matrix. The results show that LAMP assays and qPCR SYBR Green method (using the F3/B3 LAMP external primers) are equally sensitive, and they are more sensitive than conventional PCR.

The analytical sensitivity is affected by the matrix investigated. This was clear when the LoD of a DNA extract from *A. bungii* larva serially diluted 1:4 (from 10 ng/L to 2.38 fg/µL) was compared with the values resulting from the LoD of the LAMP assay on *A. bungii* 's artificial frass. The LAMP test studied was 10^3^ (from 0.61 pg/l to 9.53 fg/µL) more sensitive from the “pure” matrix of *A. bungii* larva than the corresponding artificial frass. These values confirm that the starting matrix is difficult to extract and amplify, but at the same time indicate the excellent performance of our LAMP assay.

A comparison of the data resulting from similar studies (Rizzo et al. 2020a), clearly show the greater analytical sensitivity of our new LAMP approach.

Although LAMP is a powerful method for the screening of samples and rapid responses, it may not be suitable when many validation parameters need to be estimated, as in the case of intra- or inter-lab comparisons (Panno et al. 2020). Moreover, the LAMP reaction is more prone to cross-contamination than other amplification techniques (Karami et al. 2011; Karthik et al. 2014).

The rapidity (less than 2 h) of our tests and, in the case of visual LAMP, the cheapness of the proposed protocols suggest their potential in the near future for preventing or managing outbreaks of *A. bungii* in areas with a high risk of introduction, especially if integrated with other monitoring tools such as pheromone or allelochemical traps. A decisive enhancement for making the method simpler to apply also in the field, could be a simplification of the DNA extraction from the frass matrix using a crude extract.

## Conclusions

The efficient management of a quarantine insect pest is based on detecting outbreaks as quickly as possible. Among the molecular methods, LAMP is a promising tool and more simple than the classical morphological approach, which requires intact samples and highly specialized skills. This is particularly true for xylophagous insects, where the sample collection is onerous in terms of time and costs, but also difficult due to the endophytic life of the preimaginal stages.
